# Video-feedback promotes sensitive limit-setting in parents of twin preschoolers: a randomized controlled trial

**DOI:** 10.1186/s40359-021-00548-z

**Published:** 2021-03-19

**Authors:** Saskia Euser, Claudia I. Vrijhof, Bianca G. Van den Bulk, Rachel Vermeulen, Marian J. Bakermans-Kranenburg, Marinus H. van IJzendoorn

**Affiliations:** 1 University of Leiden, Leiden, The Netherlands; 2 Erasmus University Rotterdam, Rotterdam, The Netherlands; 3 UCL, London, UK

**Keywords:** Randomized controlled trial, Parenting intervention, Video-feedback, Positive parenting, Sensitive discipline, Preschoolers, Twins, Differential susceptibility, Temperament

## Abstract

**Background:**

Primary aim of the current randomized controlled trial was to test the effectiveness of the parenting intervention ‘Video-feedback to promote Positive Parenting and Sensitive Discipline’ (VIPP-SD) in a sample of parents of preschool-aged twins, as well as differential susceptibility to intervention efforts, that is, whether more temperamentally reactive parents would profit more from the VIPP-SD than parents with lower reactivity.

**Methods:**

The sample consisted of 202 families with same-sex twins [N = 404 children, mean age 45 months (SD = 6.81)]. Randomization was done at the family level in a 2:3 ratio, with 83 families (41%) randomized to the VIPP-SD group, and 119 families (59%) to the control group. After two pre-tests in year 1 and year 2 of the study, the VIPP-SD was implemented in the third year, with a post-test assessment 1 month after the five intervention sessions. Parental sensitivity was observed during structured play in which parent and child copied a drawing together in a computerized Etch-A-Sketch paradigm. Parental limit-setting was observed in a ‘don’t touch’ task in which the parent required from the child to abstain from playing with attractive toys. Parents interacted with each of their twins in separate sessions.

**Results:**

The VIPP-SD intervention had a positive impact on the level of parents’ positive limit-setting in interaction with their preschool twins, and this positive effect was most pronounced when the parents completed at least five intervention sessions. However, the intervention did not enhance parental sensitivity during structured play. Parents with higher reactivity were not more open to the impact of the intervention, thus for this temperamental marker differential susceptibility in adults was not supported.

**Conclusions:**

The current study is unique in targeting families with twin preschoolers, providing proof of principle that coaching parents with video-feedback promotes parental sensitive limit-setting to both children. It remains to be seen whether this finding can be replicated in families with non-twin siblings, or other parental susceptibility markers.

*Trial registration* Trial NL5172 (NTR5312), 2015-07-20.

## Background

In the genomic era it has been called into question whether parents have any influence on their children beyond conception. From a behavioral as well as a molecular genetics perspective, evidence has accumulated that emphasizes the impact of genetic differences on human development as well as the influence of intractable unique experiences. Little room seems to be left for the shared environment such as parenting style to shape development [1–3]. Whereas Judith Harris in her book on ‘The Nurture Assumption: Why Children Turn Out the Way They Do’ [2] argued that the shared environment of families did not affect child development, she still left room for the shared environment of peers and neighborhoods to make a difference in developmental trajectories of adolescents. In his book on ‘Blueprint. How our DNA makes us who we are’, Robert Plomin [3] summarizes several decades of behavioral and molecular genetic research on child development, in particular intelligence and school achievement, and he is even more radically rejecting the idea that shared influences of families, schools and peers would be important. ‘Families matter but they don’t make a difference’ is the bottom line of his grand and personal synthesis of the genetics literature.

Against this background it seems to make little sense to try and change parenting to improve the development of their children. First, the development of parents might also escape shared influences and be shaped mostly by their DNA as well as volatile unique experiences. However, previous twin studies have suggested that a substantial amount of the variance in observed parenting can be explained by shared environment, with only small or non-significant child genetic effects [4–8]. Second, even if parent training or support would change their parenting style, it still may be doubtful whether such changes in the shared environment would make a difference for the children’s development. In the current paper we focus on the first question, namely whether parenting can be changed through a parent coaching program using video-feedback, and we address the question which parents are most open, i.e. susceptible to the impact of this video-feedback intervention. An outstanding question for further research remains the susceptibility of the children to (a change in) parenting.

In the current randomized controlled trial the parenting support program Video-feedback to promote Positive Parenting and Sensitive Discipline (VIPP-SD) [9, 10] has been adapted and used to test its influence on parents of preschool twins, and to examine differential susceptibility effects of the intervention on parents differing in temperamental reactivity [11–13], in particular their orienting sensitivity to external physical and social stimuli [14]. VIPP-SD is based on two research traditions [10]. Attachment theory inspired the developers of VIPP-SD to build the intervention around stimulating the parents to reflect on the sensitivity of their actions and responses to the children’s initiatives and reactions as the interactions are mirrored on video-tape [15, 16]. Social learning theory offered insights into how parents can set firm and consistent limits to their children and to avoid falling into the trap of coercive cycles from which children often emerge as victorious and the parents as their victims [17, 18].

In 12 randomized controlled trials on a large variety of typical and atypical samples (total N = 1116) the effectiveness of VIPP-SD to enhance parental sensitivity and sensitive limit-setting has been tested. Overall, the intervention program appeared to be effective in improving parenting, with a combined effect size of around half a standard deviation difference between intervention and control group [10]. VIPP-SD showed the largest effects in samples screened for insensitive base-line parenting and in poverty samples. Child outcomes, which are indirect targets of the intervention, are somewhat harder to change into a positive direction, but still the overall impact in decreasing attachment insecurity and behavior problems amounts to a small to medium effect size, also in the long run [10]. Some trials have examined differential effects on child outcomes as predicted by differential susceptibility theory [19] and found larger effects of VIPP-SD in children with a specific dopamine-system related genotype (DRD4-7repeat) [20, 21] and with a reactive temperament [22]. However, differential effects of VIPP-SD interventions on parents have not yet been studied.

Differential susceptibility theory suggests that some individuals are more affected—for better *and* for worse—by their experiences of the environment than are others, and that more susceptible individuals may be identified by their temperamental reactivity, sensitivity to context, or their genetic make-up [13]. In fact, the three main markers of differential susceptibility have in the past been considered risk or vulnerability factors that in combination with environmental adversities might lead to less optimal or even atypical development. From this cumulative risk or diathesis stress model, temperamental reactivity would, for example, lead to extreme shyness in children growing up in socially harsh environments [23]. Differential susceptibility, however, draws attention to the bright side of this interaction effect, namely a potential for benefits of the same ‘vulnerable’ individuals in supportive environments in which they would even outperform individuals with a less susceptible constitution. The implication is that parenting support programs might have stronger effects on parents who are more temperamentally reactive or sensitive to stimuli compared to parents who are less reactive, even when their base-line quality of parenting is similar. The consequence of assessing intervention effects only across the whole group is that any increased efficacy of the intervention in the susceptible subgroup might remain hidden [19] as the main intervention effects might be located in the interaction with parents’ temperament or other susceptibility markers (cf. [24]). In the current study we used the orienting sensitivity scale from the Adult Temperament Questionnaire [14, 25] to identify those parents who might be more susceptible to the environment and thus might profit more from the VIPP-SD program.

Twins have been often used as ‘guinea pigs’ in descriptive studies on the behavioral genetics of a large variety of human traits and characteristics in the domains of physical and mental health. In 2015 Polderman et al. [26] reported on a meta-analysis of 17,804 traits from 2748 publications including 14,558,903 twin pairs [26]. However, intervention studies with the aim of supporting parents of twin children are extremely rare. Nevertheless, families with twins are important targets for parent coaching as they struggle more than typical families with challenges around dividing their attention and managing potential jealousy issues between the two same-age children [27–29]. For example, when the twins are in distress they cannot always be consoled at the same time by the parent who might succeed in alleviating the distress of one child at the expense of increasing the distress in the other child. Or, as an example in the domain of limit-setting, the parent’s involvement with one of the twins to prevent them from touching a forbidden object might provide the other twin with an opportunity for noncompliance. Such competing demands on the parents might well lower their potential level of sensitivity and sensitive limit-setting, and they may profit from reflecting on video-taped interactions to better deal with such demands.

Furthermore, families with twins create unique opportunities to examine differential susceptibility of children within the same family. Originally, Belsky [11] hypothesized that parents would elevate their chances of inclusive fitness most if their offspring would vary in susceptibility to the environment. Because in evolutionary times the future was not always predictable survival chances of offspring might be promoted if some children would be more rigidly adapting to the environment that their parents experienced and foresaw for their offspring whereas siblings with more flexible adaptability would profit from unexpected changes in the environment. Variation of differential susceptibility within the same family would therefore improve inclusive fitness through hedging the parental bets on an uncertain future [11, 30]. In the current study our focus is on the intervention effects on the parents, and whether they treat their children differently. Few studies did examine differential susceptibility to the environment in adults. In a previous study some parents appeared to be more and others less impacted by daily stresses in responding sensitively to their offspring’s signals, depending on parents’ dopaminergic system genes as susceptibility marker [31]. In the same vein, parental susceptibility to environmental influences may increase the impact of a supportive parenting intervention. Additionally, families with twins can be used to examine whether intervention effects on parenting change variability between parental interactions with the two the children in the same family.

As reported in the pre-registered study protocol [32], the primary aim of the study is to test the effect of the VIPP-SD on parental sensitivity and sensitive limit-setting. The first hypothesis is that sensitivity and sensitive limit-setting of parents in the intervention condition will significantly increase post-intervention, compared to sensitivity and sensitive limit-setting of parents in the control condition. We will examine whether children within families might trigger different parenting intervention effects. Focusing on the parents, the second hypothesis of the current study is that parents who are more temperamentally reactive will profit more from the VIPP-SD than parents with lower reactivity. This is a test of one of the markers of differential susceptibility.

## Methods

### Study design

The L-CID preschooler project is a randomized controlled trial with annual assessments from age 3 onwards. Participants are families with same-sex twins living in the western region of the Netherlands. The study was planned to consist of six yearly assessments [32] but the COVID-19 pandemic might necessitate some changes [33]. Each assessment consists of a home or laboratory visit and several ambulatory assessments that are carried out by the parents at home. After two baseline assessments in Wave 1 and Wave 2, a random 40% of the sample received an intervention aimed at enhancing parental sensitivity and sensitive discipline strategies of the primary caregiver, the VIPP-SD [9, 10], that started 8 months after the Wave 2 assessment. The split was chosen to be 40–60% instead of the usual 50–50% because of the limited resources to conduct the VIPP-SD intervention sessions with trained interveners within a relatively small time window [32]. The first post-test assessment (Wave 3) was carried out 1–2 months after the intervention, i.e. 1 year after Wave 2, and for the current study data from the two baseline assessments and this first post-test assessment of the primary parent were used (Waves 1, 2 and 3). The first assessment was a home visit, and for the second and third assessments families were invited to the laboratory. Parental sensitivity and limit-setting were measured at each assessment using the Etch-a-Sketch task and a Don’t touch task. Before the first assessment, both parents/legal guardians gave written informed consent. The research protocol was approved by the Central Committee on Research Involving Human Subjects in the Netherlands (CCMO; NL49069.000.14). The design of the current intervention study has previously been described in detail in a pre-registered study protocol [32]. The study adheres to CONSORT guidelines.

### Participants

#### Recruitment

Families with twins were selected from municipality records with the following criteria: twins had the same sex, and their parents and grandparents were born in Europe. Mental or physical disorders such as congenital disability, psychological disorder, chronic illness, hereditary disease, or a visual or hearing impairment were reason to exclude children as they would likely make the child unable to participate in the study tests and tasks, some of which were based on EEG and MRI. Intellectual disability (IQ < 70) was an exclusion criterion for the same reason.

Eligible families (*n* = 871) received an invitation letter and information brochure by mail. In a subsequent phone call, research assistants checked the inclusion criteria with the parents who were willing to participate, and they were then invited for the first home visit. Parents received a financial reimbursement of €60 after home visits and €80 after laboratory visits, children received annual gifts, and travel expenses were compensated.

#### Study sample

**Fig. 1:**
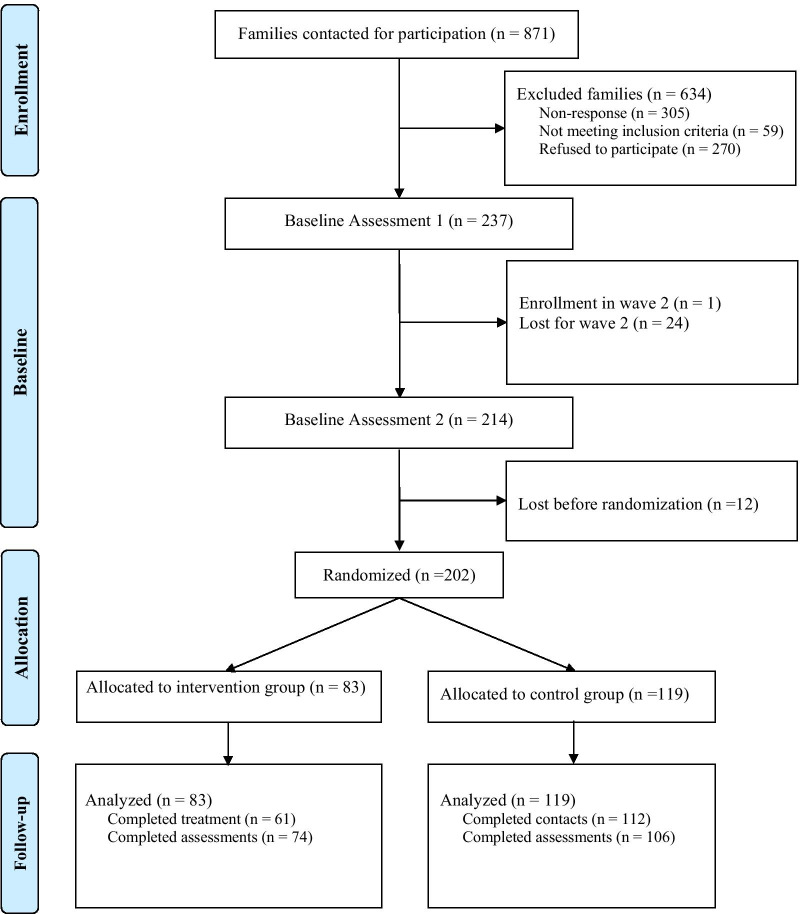
Flow chart of the sample recruitment and randomization in the trial

In total, 871 families received an invitation letter. More than a third of the families (35%) did not respond to the invitation letter, 7% of the families did not meet the inclusion criteria, and 31% of the families did not want to participate. Thus, 27% of the families were enrolled in the study, leading to a sample of N = 237 families, each with 2 twin children (N = 474 children, 58% monozygotic pairs) for the first baseline assessment. Recruitment and randomization of the families are shown in a flow chart (see Fig. 1). Due to privacy regulation no information on families who did not respond to the invitation letter was available. However, for declining families some information was available, and they did not differ from participating families on any of the background characteristics (see [32] for details). At the time of recruitment, participating twins were on average 3.6 years old (SD = .57), and 50% were boys. After the first baseline assessment, one more family responded to the invitation letter and was enrolled in the second baseline assessment. A small number of participants decided not to be involved in the first two assessments or in the randomization process which led to a final sample of *n* = 202 families who were included in the analyses of the current paper (see Fig. 1). Sample characteristics of the final sample are shown in Table 1. It should be noted that the external validity or generalizability of the results might be impacted by the relatively low percentage of positive responders but that the internal (conclusion) validity is not influenced because the attrition took place before randomization [34].

**Table 1:** Sample characteristics, separately for intervention and control condition

	Total (N = 202)	Intervention group (n = 83)	Control group (n = 119)
Twin characteristics			
Age at baseline 1 in months M (SD)	45.07 (6.81)	44.89 (7.44)	45.20 (6.37)
Gender (% boys)	45	45.8	44.5
Country of birth (% Netherlands)	99.5	100	99.2
Family characteristics			
Primary parent (%)			
Biological mother	91.6	88.0	94.1
Biological father	8.4	12.0	5.9
Age primary parent M (SD)	36.87 (4.69)	36.89 (4.82)	36.82 (4.62)
Age second parent M (SD)	38.49 (5.64)	38.39 (5.69)	38.56 (5.62)
Country of birth (% Netherlands)			
Primary parent	96.0	98.8	94.1
Second parent	96.4	97.5	95.6
Educational level primary parent			
Primary school	0.5	1.2	0
Lower vocational education	4.0	4.8	3.4
Higher general secondary education, pre-university education, intermediate vocational education	25.7	31.3	21.8
Higher vocational education, BA	42.1	36.1	46.2
MA, postgraduate level	27.7	26.5	28.6
Family SES			
Low	6.4	7.2	5.9
Medium	38.6	42.2	36.1
High	55.0	50.6	58.0
Number of children in the family M (SD)	2.80 (.76)	2.87 (.79)	2.75 (.74)
Primary parents’ marital status (%)			
Married or registered partnership	70.3	63.8	74.8
Cohabiting	26.2	32.5	21.8
Single parent	3.5	3.6	3.4
Family type (%)			
Biological parent(s)	97.5	98.8	96.6
Parental reactive temperament M (SD)	4.31 (.72)	4.23 (.67)	4.37 (.75)
Parental psychopathology symptoms Wave 1	1.26 (.29)	1.21 (.24)	1.30 (.32)*
Parental psychopathology symptoms Wave 2	1.30 (.34)	1.24 (.26)	1.34 (.38)*
Parental psychopathology symptoms Wave 3	1.28 (.29)	1.22 (.24)	1.33 (.31)*

^*^Significant difference between the intervention and control condition (*p* < .05)

### Intervention

#### Randomization

Randomization to the VIPP-SD or control condition was done at the family level in a ratio of 2:3, using a computer-generated blocked randomization sequence, with a block size of 19 families based on timing of the intervention and stratified by parent and twin sex. 83 families (41%) were randomized to the intervention group, and 119 families (59%) to the control group. As noted above, we had to restrict the number of families participating in the intervention because of limited resources, with only marginal loss of statistical power. Randomization was performed after the second baseline assessment, right before the start of the intervention, in order to prevent selective attrition. Assignment of participants was performed by an independent researcher who was not involved in data collection or coding and used a random numbers generator. Researchers, interveners and participants were blinded to randomized assignment, but not after this assignment to intervention or control group, because of the open-label design. To minimize bias based on knowledge about allocation of participants, coders and research assistants who carried out the post-test assessments were blind to treatment allocation. Of course, the same was true for the pre-tests.

#### VIPP-SD for twins

Video-feedback Intervention to promote Positive Parenting and Sensitive Discipline (VIPP-SD adapted to twin families) was implemented between Wave 2 and Wave 3 (see Fig. 1). VIPP-SD was slightly adapted for the use with twin families in the current study. Parenting twins may lead to challenges for parents, such as dividing attention and sharing or competition between twins, which are less relevant for parents with singletons. See [32] for more details on these adaptations.

In short, the VIPP-SD consists of five biweekly sessions in which families are visited at home by a female intervener. All interveners were extensively trained in implementing the intervention by using the standardized manual describing the structure, themes, tips, and exercises for parent and children for each session (manual VIPP-SD version 3.0) and subsequently trained in the specific focus on parents of twins [35]. Every session started with videotaping approximately 15 min of standardized parent–child interactions, such as playing or reading a book together [20]. Between sessions, the intervener prepared comments on the child’s or parent’s behavior based on the theme of the next session and selected illustrating video fragments. In the next session, after collection of new video material, the video of the previous session was reviewed with the parent and video feedback was given using the relevant video fragments. During this feedback period, the intervener focused on positive and successful interaction moments and indicated moments of effective positive parenting [10].

The first theme session focused on exploration versus attachment behavior, highlighting the difference between the children’s play and proximity seeking, and the differential parent responses needed. Distraction and inductive discipline as non-coercive responses to difficult child behavior were introduced. During the second session, attention was drawn to the perception of the children’s (subtle) signals, using ‘speaking for the child’, and to the use of positive reinforcement by praising positive child behavior and ignoring negative attention seeking. In the third session, the importance of prompt and adequate responding to the children’s signals was explained by showing positive interaction chains. In addition, the use of a sensitive pause to de-escalate temper tantrums was discussed. The themes of the fourth session were sharing emotions, showing the parent the importance of attunement to both positive and negative emotions of their child, and promoting empathy for the child during consistent and adequate limit-setting strategies. In the first four intervention sessions, only the primary parent was present. These are followed by a booster session, in which all themes were repeated and integrated. The parents’ partner was invited to participate in the final session according to protocol, in order to enhance his or her empathy and support of the primary parent’s implementation of changes in parenting. Interveners kept logs about adherence to the intervention protocol which were used for regular intervision and fidelity checks.

#### Control condition

To ensure that the control families would receive the same number of contacts with the research team, these families received five phone calls from a research assistant parallel to the intervention sessions. The phone calls followed a standard protocol of a semi-structured interview about the general development of their twins used in previous randomized controlled trials. No specific information or advice about parenting or child development was provided (e.g. [20]).

### Measures

#### Sensitivity

Parental sensitivity was observed for both co-twins separately during a structured play situation. The primary parent, in most cases the biological mother (see Table 1), performed the task twice, once with each of the twin siblings. The order (oldest child first, youngest child first) was random across families. In a computerized version of the Etch-A-Sketch task [36], the parent–child dyads were instructed to make three drawings on a computer screen, following printed examples with increasing difficultness (see [32] for details of the task). The duration of the task was 10 min in the first assessment and 8 minutes in the second and third assessments, when they were already familiar with the equipment and procedure. Four minutes after the start of the game (3 min in the second and third assessments), an audio sign instructed the participants to start with the second drawing if they had not done so already. Parent–child interaction was filmed and the drawing on the screen was recorded. A single video was created with both recordings side by side.

Parental sensitivity was coded using the revised Egeland et al. [37] 7-point rating scales for supportive presence (1 = parent completely fails to be supportive to the child, 7 = parent skillfully provides support throughout the session) and intrusiveness (1 = parent allows the child sufficient time to explore and to attempt to solve tools on her/his own, 7 = parent is highly intrusive; her/his agenda clearly has precedence over the child’s wishes [37, 38]). The videos were coded by fourteen coders, trained by an expert coder (SE). Intercoder reliability (intraclass correlation coefficient; ICC) with the expert coder and among coders was adequate. For wave 1 (n = 47 tapes; five coders), the mean ICC for supportive presence with the expert coder was .82 (range .79–.85) and among coders .77 on average; for intrusiveness the mean ICC with the expert coder was .80 (range .74–.85) and among coders .74 on average. For wave 2 (n = 40 tapes; 5 coders), the mean ICC for supportive presence with the expert coder was .83 (range .76–.89) and among coders .83 on average; for intrusiveness the mean ICC with the expert coder was .77 (range .72–.81) and among coders .79 on average. For wave 3 (n = 48 tapes; 6 coders), the mean ICC for supportive presence with the expert coder was .74 (range .68–.77) and among coders .71 on average; for intrusiveness the mean ICC with the expert coder was .75 (range: .68–.80) and among coders .76 on average. Videos of co-twins or from the same family in two different waves were never coded by the same coder.

Intrusiveness scores were recoded so that higher scores on both scales indicated higher parental sensitivity. The correlation between the two scales ranged from *r* = .53 to *r* = .65 across twins and study waves. The scores for supportive presence and intrusiveness were combined into a single measure of sensitivity by taking the mean of the two scores. The final sensitivity variable was normally distributed without outliers.

#### Sensitive limit-setting

Parental limit-setting was observed in a don’t touch task [39] (see [32] for details). Parents performed this task twice, once with each child. The order of the children was random between families. Parents received written instruction explaining the task before they were handed a bag of attractive toys. They were requested to take all the toys out of the bag, and to tell the child not to touch any of the toys. After 2 min, the child was allowed to play with the least attractive toy only. This episode also lasted 2 min. They were then allowed to play with all of the toys for a few minutes (this episode was not coded). The task was video-taped and parental limit-setting was coded by fourteen coders, including an expert coder (CV) who trained the other coders.

Two scales were used: positive discipline, rated on an adapted version of the revised Erickson 7-point rating scales for supportive presence (1 = parent completely fails to provide positive discipline, 7 = parent skillfully provides positive discipline throughout the session [37], and physical interference, rated on a 5-point scale (1 = parent does not interfere physically, 5 = parent often interferes physically; see [40] for the adaptation). Intercoder reliability (intraclass correlation coefficient; ICC) with the expert coder and among coders was adequate. For wave 1 (n = 50 tapes; five coders), the mean ICC for positive discipline with the expert coder was .77 (range .71–.80) and among all coders .76 on average. For wave 2 (n = 48 tapes; four coders), the mean ICC for positive discipline with the expert coder was .74 (range .71–.79) and among all coders .76 on average. For wave 3 (n = 50 tapes; seven coders), the mean ICC for positive discipline with the expert coder was .79 (range .73–.88) and among all coders .81 on average. Videos of co-twins or from the same family in two different waves were never coded by the same coder.

The physical interference scale was also scored with high inter rater reliability but it showed a skewed distribution with low variance as physical interference characteristic of the higher scores was rarely shown. Furthermore, the correlation between positive discipline and physical interference was low, and we decided to use only the positive discipline scale for the current analyses. Scores on sensitive limit-setting were approximately normally distributed, without outliers.

#### Primary parent temperamental reactivity

Before the post-test assessment parents reported on their own and their partner’s temperament, using the 15-item Orienting Sensitivity scale from the Adult Temperament Questionnaire short form [14, 25]. The scale consists of three subscales, neutral perceptual sensitivity (detection of slight, low intensity stimuli from within the body and external environment), affective perceptual sensitivity (spontaneous emotionally valenced, conscious cognition associated with low intensity stimuli), and associative sensitivity (spontaneous cognitive content that is not related to standard associations with the environment). We decided to label the combined sub-scales ‘temperamental reactivity’ to avoid confusion with observed parental sensitivity to their children, and to emphasize this temperamental feature as a marker for differential susceptibility. Example items are “I often notice mild odors and fragrances”, “When I watch a movie, I usually don’t notice how the setting is used to convey the mood of the characters” (reversed), and “I sometimes seem to understand things intuitively”. Items were answered on a 7-point Likert scale ranging from extremely untrue to extremely true. In addition, participants could answer ‘not applicable’ when the statement did not apply to them or their partner, and these answers were coded as missing. Four items were reverse coded, so that higher scores indicated higher temperamental reactivity. Reliability for the self-report and partner report were high (Cronbach’s *α* = .85 and .80 respectively). Scores for self-reported and partner-reported temperamental reactivity correlated significantly (*r* = .39) and were combined in a single measure by taking the mean of the two scores. If either self- or partner-reports were missing, the available score was used as measure of parental temperamental reactivity. There were no data available for 37 families (18%), and these values were imputed using the EM option in SPSS, with age and gender of the primary parent, age and gender of the twin, parental educational level, and parental psychopathology symptoms as predictors. The imputed variable was normally distributed without outliers, highest score was 7.

#### Primary parent psychopathology

To control for possible differences in symptoms of psychopathology, the primary parent completed four subscales of the Dutch version of the Brief Symptom Inventory (BSI) [41, 42] prior to each assessment. They reported on symptoms they experienced in the past week for depression (6 items), anxiety (6 items), hostility (5 items), and interpersonal sensitivity (4 items). Items were answered on a five-point scale (0 = not at all, 4 = a lot). The reliability of the combined scales was good (*α* = .88 in wave 1; *α* = .91 in wave 2; *α* = .84 in wave 3). An average score was computed based on all 21 items, with higher scores indicating more psychopathology symptoms. No data were available for 11 families (5%) in wave 1, for 29 families (14%) in wave 2, and for 42 families (21%) in wave 3. Missing values were imputed using the EM option in SPSS, with age and gender of the primary parent, age and gender of the twin, parental educational level and psychopathology symptoms in subsequent years as predictors. Log transformations of the scores were performed to approach a more normal distribution of the data.

### Statistical analyses

First, we examined differences between the intervention and control group on background variables. Background variables on which the two groups differed significantly were included as covariates in subsequent analyses. Means and standard deviations of the outcome variables were computed and correlations within twins and across time were explored.

The effects of the VIPP-SD on parental sensitivity and parental sensitive limit-setting were analyzed using intent-to-treat analyses. Because of the longitudinal design of the study and the nested structure of the data of twins within families, we used multilevel analyses (mixed models in SPSS 25.0) with full maximum likelihood estimation. The data consisted of three levels (time of assessment, child, family), and intraclass correlation coefficients (ICCs) were calculated to examine the proportion of variance on each level. Levels were not included in further analyses if the ICC was not significant or < .05 [43]. Two separate models were tested for the two outcome variables, parental sensitivity and parental sensitive limit-setting. First, we fitted an intercept only model with three levels and an unrestricted within-subject (co)variance structure to compute the ICC. Next, we fitted a growth model with time and time squared added as fixed and random effects. Time was coded as − 1, 0, 1, so that effects on the intercept could be interpreted as differences at baseline 2. We were interested in the quadratic growth over time, because the intervention occurred between the second and third assessment. We expected a stronger quadratic time effect in the intervention group compared to the control condition. As covariates we included parental psychopathology symptoms, because the intervention and control groups differed significantly on this variable at pretest, and educational level, because it was significantly associated with the parenting variables. Condition (control or intervention group) and the interaction between condition and time^2^ as fixed effects were added to the model in order to test the main intervention effect. Condition was coded as 0 = control and 1 = intervention. A significantly higher condition*time^2^ effect in the intervention group would indicate a significant effect of the VIPP-SD on sensitivity or sensitive limit-setting. The three-way interaction with time^2^, condition, and parental temperamental reactivity was added to the model as fixed effect to examine the pre-registered, hypothesized moderating role of reactivity as a marker of differential susceptibility. All predictor variables were standardized before inclusion, so multivariate statistics can be interpreted as standardized coefficients (see Tables 3 and 4).

We performed two robustness analyses. First, we tested the same model in families who received all five intervention sessions or five telephone calls in the control condition. Second, we tested the model in families with complete assessments, excluding families with missing data on the outcome variable on any of the three assessments (complete cases analysis).

The a priori power of the statistical analyses was excellent (> 90%) for the primary hypothesis, assuming a previously established meta-analytic effect size of d = .47, with α = .05 and a sample size of 225 families, including 450 children (see [32]).

## Results

### Preliminary analyses

Sample characteristics are shown in Table 1 for the total group, and for the intervention and control group separately. Randomization led to equal scores  for most background variables, except for parental psychopathology; parents in the control condition reported significantly more psychopathology symptoms compared to parents in the intervention condition in all three waves. Scores on parental psychopathology symptoms were included as covariate in the analyses. Intervention and control groups did not differ on parental temperamental reactivity. Overall treatment integrity was high, 173 families (86%) received the complete VIPP-SD intervention or ‘dummy’ contacts.

Descriptive statistics of the outcome variables and parental temperamental reactivity are shown in Tables 1 and 2. Within-twin and across time correlations were significant for both co-twins and all assessment waves. For parental sensitivity, within-twin correlations ranged between *r* = .59 and *r* = .62 for the different waves and correlations across time ranged between *r* = .40 and *r* = .60 for the two children and different waves. Within-twin correlations were comparable for parental sensitive limit-setting, ranging between *r* = .56 and *r* = .60 for the different waves, but across-time correlations were somewhat lower, ranging between *r* = .19 and *r* = .47 for the two children and different waves (see Euser et al. [5] for a behavioral genetics analysis).

**Table 2:** Descriptive statistics of outcome variables at baseline 1, baseline 2, and post-test assessment

	T0		T1		T2	
	Child 1	Child 2	Child 1	Child 2	Child 1	Child 2
Parental sensitivity						
Intervention group	3.89 (1.41)	3.76 (1.33)	3.84 (1.41)	3.88 (1.42)	4.11 (1.50)	3.96 (1.36)
Control group	3.98 (1.27)	4.07 (1.28)	4.10 (1.44)	4.26 (1.34)	4.24 (1.30)	4.35 (1.35)
Parental sensitive limit-setting						
Intervention group	4.97 (1.37)	4.96 (1.34)	4.92 (1.28)	4.72 (1.36)	4.84 (1.34)	4.92 (1.18)
Control group	5.41 (1.16)	5.56 (1.22)	5.27 (1.32)	5.34 (1.19)	4.98 (1.32)	5.14 (1.33)

### Intervention effects on parental sensitivity

In the intercept only model with three levels, the ICC indicated that 51% of the variance in parental sensitivity could be accounted for by the family, whereas the variance at the child level was zero. Therefore, only time and family were included as levels in the further steps. Table 3 shows multivariate statistics for the multilevel analysis testing the VIPP-SD main effect and the reactivity moderator effect on parental sensitivity.

**Table 3:** Multivariate statistics of multilevel analysis testing the intervention effect on parental sensitivity

				95% confidence interval	
Predictor	Estimate	Std. error	Sig	Lower bound	Upper bound
Intercept	3.96	.11	.000	3.74	4.17
Time	.07	.11	.539	− .15	.28
Time^2^	.03	.11	.784	− .19	.26
Education	.34	.07	.000	.20	.48
Psychopathology	− .04	.05	.468	− .14	.07
Condition	.17	.14	.243	− .11	.45
Condition*Time^2^	.02	.07	.785	− .12	.15
Reactivity	.06	.12	.581	− .16	.29
Condition*Reactivity	.08	.14	.569	− .20	.37
Reactivity*Time^2^	.03	.06	.618	− .08	.14
Condition*Reactivity*Time^2^	− .08	.07	.247	− .21	.06

The interaction between condition and quadratic time was not significant (*β* = .02, *p* = .785). Parents in the intervention condition did not show more quadratic growth over time compared to parents in the control condition, suggesting that the intervention did not support the parents in enhancing their sensitive parenting shown over time. Neither the main effect of temperamental reactivity (*β* = .06; *p* = .581), nor the moderation by parental temperamental reactivity of the intervention effect to test for possible differential susceptibility was significant (*β* = − .08; *p* = .247). Without covariates the results were similar.

#### Robustness analyses

The robustness analysis including only families with complete treatment or only complete cases did not show condition*time^2^ effects or moderation effects of parental temperamental reactivity and thus converged with the main analyses.

### Intervention effects on parental sensitive limit-setting

The ICC in the intercept only model with all three levels indicated that 40% of the variance in parental limit-setting could be accounted for by the family level, whereas none of the variance could by accounted for by the child level. The child level was therefore not included in the analysis. Table 4 shows multivariate statistics for the multilevel analysis testing the VIPP-SD main effect and reactivity moderator effect on parental sensitive limit-setting.

**Table 4:** Multivariate statistics of multilevel analysis testing the intervention effect on parental sensitive limit-setting

Predictor	Estimate	Std. error	Sig	95% confidence interval	
				Lower bound	Upper bound
Intercept	4.93	.10	.000	4.73	5.12
Time	− .18	.12	.134	− .43	.06
Time^2^	.16	.12	.191	− .08	.40
Education	.20	.06	.002	.08	.33
Psychopathology	.07	.05	.167	− .03	.18
Condition	.34	.13	.009	.09	.59
Condition*Time^2^	− .15	.07	.046	− .29	− .00
Reactivity	− .00	.10	.974	− .21	.20
Condition*Reactivity	.00	.13	.996	− .25	.26
Reactivity*Time^2^	.06	.06	.289	− .05	.18
Condition*Reactivity*Time^2^	− .06	.07	.446	− .20	.09

**Fig. 2:**
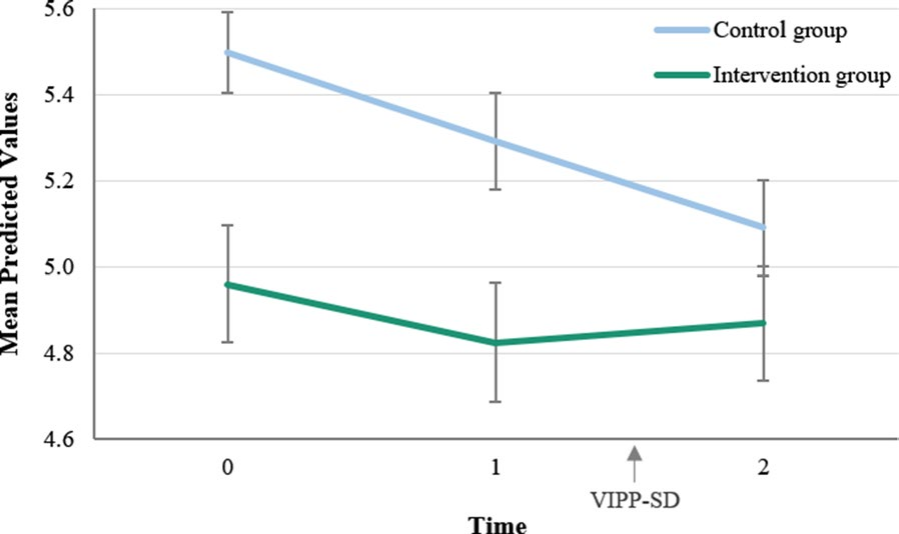
Intervention effects on parental sensitive limit-setting (mean predicted values, standard errors; N = 202 families with twins)

The effect of condition showed that parents in the control condition scored significantly higher on sensitive limit-setting on pretest 2 (the intercept: *β* = .34; *p* = .009). However, the interaction between condition and quadratic time was significant (*β* = − .15, *p* = .046). Parents in the intervention condition showed more quadratic growth (or less decline) over time compared to parents in the control condition, suggesting that the intervention supported these parents to level off the decline of sensitive limit-setting shown over time by the parents in the control condition. Predicted values of the condition*time^2^ effect are graphically shown in Fig. 2. Finally, the moderation by parental temperamental reactivity of the intervention effect was not significant (*β* = − .06; *p* = .446). Without covariates results were similar.

#### Robustness analyses

The robustness analysis including only families with complete sessions (N = 173) showed a more pronounced interaction effect between condition and quadratic time (*β* = − .22, *p* = .006). Parental temperamental reactivity as a moderator did not significantly explain variance in sensitive limit-setting (*β* = − .09, *p* = .275). The robustness analysis including only families with complete cases (N = 175) showed a similar effect of the intervention as the intent-to-treat analysis (*β* = − .16, *p* = .033), and again no moderator effect of parental reactivity (*β* = − .07, *p* = .323).

## Discussion

The VIPP-SD intervention had a positive impact on the level of parents’ sensitive limit-setting in the interaction with their preschool twins, and this effect was most pronounced when the parents completed most or all of the intervention sessions. In the control group the parents had increasingly more problems with setting limits in a ‘don’t touch’ task in which the child was required to abstain from playing with attractive toys, whereas in the intervention group this downward trend was leveled off as the parents had learnt how to set firm limits in a sensitive way. However, the intervention did not enhance parental sensitivity in structured play (copying drawings together using a computerized Etch-A-Sketch) requiring active participation of the parents but not limit setting. Against our expectation, no differential susceptibility was revealed: parental sensitivity and limit setting were not impacted most by the VIPP-SD program in the group of parents with higher temperamental reactivity. Intervention effects of parenting to each of the twin children in the same family were similar, suggesting that VIPP-SD impacts limit-setting on the family level, and thus to the same extent to each of the twins.

Addressing the doubts about the influence of coaching programs for parents in the genomic era, the current randomized controlled trial demonstrates the possibility of coaching using video-feedback to change parental limit setting for the better. Our experimental intervention study shows the efficacy of VIPP-SD in particular when parents participate in all sessions of the program (86% of them did). Participating in all sessions enhanced the efficacy of the VIPP-SD by half (from an effect size of − .15 to − .22). VIPP-SD enhances parental sensitive limit-setting with a medium effect size –according to the conventional Cohen criteria—of *β* = .22, comparable to a Cohen’s d of about .45. This effect size is close to the combined effect size of 12 randomized controlled trials with VIPP-SD (d = .47) [10].

Cohen [44] however did not recommend his criteria for small (d = .20), medium (d = .50), and large (d = .80) effect sizes to be used as universal conventions for all scientific domains of inquiry. Instead, he urged to use domain-specific criteria to evaluate the strength of the evidence emerging from a specific study. Following this advice, Kraft [45], for example, developed benchmarks for effect sizes in educational intervention research based on randomized interventions targeting student achievement with standardized test outcomes. He found that Cohen’s d less than.05 should be called small, from .05 to less than .20 would be medium, and d = .20 and above should be considered large. Our study is situated in the developmental domain and according to the analysis of meta-analyses in Psychological Bulletin [46] a medium effect size in this field would be around d = .26 (Supplement, Stanley et al. [46]). Against these domain-specific yardsticks our effect size is substantial and goes above a medium effect.

Nevertheless, one might argue that the effect of *β* = .22 explains only about 5% of the variance in post-test sensitive limit-setting. This seems a small percentage of the variation in parenting style which leaves a large part of the variance untouched. But if small effects can be transferred to large populations, they might be extremely valuable, for the individual parents as well as for society. The VIPP-SD belongs to the category of parent coaching programs with a relatively short duration and modest investments in equipment and training of interveners. Of course, booster sessions at a later stage in development might sustain and strengthen these improvements but research did not yet address this outstanding question.

Contrary to our expectations, VIPP-SD did not show the expected effect on parental sensitivity. One interpretation of this lack of effectiveness on parental sensitivity is the older age of the participating children. The VIPP-SD was originally developed for the ‘terrible twos and threes’ and the current extension into the age of 5–6 years old children might assume less need for support of parents’ sensitive interactions and more need to focus on demanding situations in which limits have to be firmly set. The high adherence to the intervention (86% of the families participated in all sessions) attests to the motivation of parents to get video-feedback, although we did not measure motivation for different parts of the VIPP-SD. Another reason might be the relatively typical development of the families and the children in the current study, who did not struggle with clinical issues and did not show a decline of parental sensitivity across time. Parents and children from adverse backgrounds might provide more room for improvement of parental sensitivity. With lower starting levels of sensitive interactions, such as observed in the study on high-risk families in which the current scales were developed [37] it is both easier and more necessary for parents to benefit in terms of parental sensitivity.

The child level did not contribute to the variance in the models we tested. This does not mean that twins in general might make it easier or more difficult for interventions to be effective, but it suggests that there are no individual differences in intervention effects on the parents for the two children within their family. For example, first-born versus second-born twin children might have experienced divergent environments in utero and show different behavior patterns after birth (for extreme cases, see Lopriore et al. [47]). Dizygotic twins, potentially phenotypically more different than monozygotic twins, may also trigger different parenting behavior and differential intervention effects of the VIPP-SD program. The level of the individual children, however, did not contribute to the explained variance in intervention effects on parental sensitivity or sensitive limit-setting. Our findings converge with several previous non-intervention studies indicating that the majority of the variance in observed parenting behaviors can be explained by shared environmental factors, with only small or non-significant child genetic effects [4–8]. For example, Euser et al. [5] reported a behavioral genetics analysis of parental sensitivity and limit-setting on the pretest data of the current study (Wave 1 and Wave 2), showing little child-based genetic influences on parenting. The VIPP-SD intervention impacted parental sensitive limit setting of both children in the family in a similar way. We may, however, not generalize this outcome to families with siblings who differ in age and developmental stage, which is an important question for future randomized controlled trials with families with siblings who are different in age and sex.

Differential susceptibility theory received support from a large number of empirical studies on temperament [48] on biological reactivity to stressful contexts [49], and on genetic markers of susceptibility [19]. In particular experimental evidence is important because of its improved statistical power to demonstrate replicable interactions between susceptibility marker and environment, for example parenting, and developmental outcome [19]. In the current study the power to find a significant moderation of temperamental reactivity was at least .80 [32]. However, we failed to find more than a weak signal that parents with higher temperamental reactivity would show more openness to the influence of the VIPP-SD resulting in a steeper increase in sensitive limit-setting. Of course, the self-report temperamental reactivity measure might be less valid for assessing reactivity in adults. Furthermore, differential intervention effects may become visible in follow-up assessments. More work is needed, in particular with more valid multi-informant assessments of temperamental reactivity and with other, potentially more valid markers of differential susceptibility on the level of genetics and neural reactivity [33]. Most studies on differential susceptibility are conducted with children and adolescents, and it may be speculated that in adulthood with smaller windows of accelerated neurobiological development differential susceptibility might be less visible. The current study holds the promise of follow-up measures of differential intervention effects in the years to come.

Some strengths and limitations of the present study should be mentioned. The intervention was pre-registered, conducted in a rather large sample, with state-of-the-art observational outcome measures of parenting [5], and included two pre-tests that allowed for stable base-line assessments against which the effects of the intervention could be evaluated. Because of the availability of pretests, we also could detect significant differences between experimental and control groups before the intervention was administered, despite careful randomization. The two groups differed on the pretests in terms of psychopathology symptoms and baseline level of parenting. The control group showed more psychopathology symptoms than the intervention group, whereas the intervention group displayed less sensitivity and sensitive limit-setting than the control group before VIPP-SD started. Such differences are unfortunate because they have to be statistically controlled for and reduce the power of the analyses. But in the absence of a pretest such unmeasured differences might lead to invalid conclusions [50].

Concerning future directions, data-collection is ongoing in this cohort-sequential randomized controlled trial in families with twins [33], and follow-up assessments of parenting might document long-term (sleeper) effects of the intervention. Furthermore, in addition to promising findings on genetic markers of differential susceptibility using candidate genes such as the serotonin transporter gene or the dopamine D4 receptor gene [19], the emergence of polygenic susceptibility scores might open broader opportunities to test genetic differential susceptibility, in parents as well as children. Using the polygenic scores developed in Eley’s team [51] for susceptibility to therapeutic intervention (see [52]) or polygenic scores associated with the dopamine system [53] as markers we may examine their interaction with the intervention efforts similar to the moderator test of temperamental reactivity in the current study. Another exciting avenue for further work is of course the study of the effects of VIPP-SD on child development, as the positive changes in parental limit-setting are expected to translate into lower levels of externalizing behaviors and better behavioral control [10, 32, 54, 55].

## Conclusions

The findings of the current randomized controlled trial do suggest that parents of twins become more sensitive in the way they set limits to their preschoolers after coaching with the video-feedback program VIPP-SD. Parents profit from this intervention that makes them aware of and reflect on subtle signals of distress, frustration or anger in their children and how to prevent or respond to the challenge of coercive cycles [10, 17]. As Hrdy [56] demonstrated for various non-human species, mammals with closely spaced births or twin births require more parent support than species with larger time delays between subsequent births. Parent coaching programs such as VIPP-SD may provide some of this support for parents coping with the stressful conditions that accompany densely stacked offspring and hamper parents in showing their full parenting potential.

## Data Availability

The data that support the findings of this study are available from the corresponding author upon reasonable request.

## Abbreviations

VIPP-SD: Video-feedback to promote Positive Parenting and Sensitive Discipline
L-CID: Leiden-Consortium for Individual Development
CCMO: Central Committee on Research Involving Human Subjects in the Netherlands
EEG: Electroencephalogram
MRI: Magnetic resonance imaging
IQ: Intelligence quotient
ICC: Intraclass correlation coefficient
EM: Expectation-maximization
SPSS: Statistical Package for the Social Sciences
BSI: Brief symptom inventory
